# The Impact of *PPARD* and *PPARG* Polymorphisms on Glioma Risk and Prognosis

**DOI:** 10.1038/s41598-020-60996-2

**Published:** 2020-03-20

**Authors:** Xiaoying Ding, Xinsheng Han, Haozheng Yuan, Yong Zhang, Ya Gao

**Affiliations:** 1 Department of Anesthesia, The Second Affiliated Hospital of Xi’an Jiaotong University, Xi’an, Shaanxi 710004 China; 2 Department of Pediatric Surgery, The Second Affiliated Hospital of Xi’an Jiaotong University, Xi’an, Shaanxi 710004 China

**Keywords:** Cancer, Biomarkers

## Abstract

Recent studies showed that peroxisome proliferator-activated receptors (PPARs) had effects on the progression of multiple tumors, but the role of *PPARD* and *PPARG* in glioma remains poorly understand. We conducted a case-control study to investigate the association of polymorphisms in *PPARD* and *PPARG* with glioma risk and prognosis in the Chinese Han population. Seven polymorphisms (*PPARD*: rs2016520, rs67056409, rs1053049 and rs2206030; *PPARG*: rs2920503, rs4073770 and rs1151988) were genotyped using the Agena MassARRAY system in 568 glioma patients and 509 healthy controls. The odd ratios (OR) and 95% confidence interval (CI) were calculated to assess the association of *PPARD* and *PPARG* polymorphisms with glioma risk. The Multifactor dimensionality reduction (MDR) method was used to analysis interactions of genetic polymorphisms on glioma risk. Then, we conducted log-rank test, Kaplan-Meier analysis and Cox regression model to evaluate the relationship of *PPARD* and *PPARG* polymorphisms with glioma prognosis. We found *PPARD* polymorphisms (rs2016520, rs67056409, rs1053049) were significantly associated with glioma risk in multiple models (*P* < 0.05). Stratified analysis showed rs2016520, rs67056409, rs1053049 of *PPARD* significantly decreased risk of glioma in the subgroup of age > 40 and astrocytoma (*P* < 0.05). For male, *PPARD* rs1053049 had a strong relationship with glioma risk in allele (*P* = 0.041), dominant (*P* = 0.040) and additive (*P* = 0.040) models. The effect of *PPARG* rs2920503 on glioma risk was related to glioma grade (*P* < 0.05). MDR showed that a seven-locus model was the best polymorphisms interaction pattern. Moreover, surgery and chemotherapy had strongly impact on overall survival and progression free survival of glioma patients. Our findings suggested that *PPARD* and *PPARG* polymorphisms were associated with glioma risk and prognosis in the Chinese Han population, and further studies are need to confirm our results.

## Introduction

Glioma is the most common type of malignant brain tumors in the central nervous system (CNS), accounting for approximately 80% of primary brain tumors^1^. The incidence of brain cancer is the highest in European (5.5/100,000 persons), North America (5.3/100,000 persons), Australia (5.3/100,000 persons), Western Asia (5.2/100,000 persons) and Northern Africa (5.0/100,000 persons)^2^. In China, there were 1,016,000 newly diagnosed cases of brain and CNS tumor in 2015^3^. Glioma occurs varied in age, sex, race, histologic type and geographic characteristics^4^. And, glioma has poor overall survival (OS), with less than 5 year survival of patients after diagnosis^5^. The etiology of glioma is multifactorial, which is the results of environmental exposure and genetic factors^4^. Single nucleotide polymorphism (SNP) is the most studied mutations involved in genetic predisposition of glioma. Recently, increasing studies are focused on the role of Peroxisome proliferator-activated receptors (PPARs) polymorphisms on cancer.

PPARs is a subfamily of nuclear receptor transcription factors and consists three isoforms (PPARα, PPARδ and PPARγ). *PPARD* encodes PPARδ, a nuclear hormone receptor that implicated in varieties of biological processes, including epidermal cell proliferation, migration, lipid and glucose metabolism^6–8^. *PPARD* is highly expressed in brain, heart, skeletal muscle, adipose tissue and pancreatic islets^9^. In mice, *PPARD* agonists increase leptin secretion and improve type 2 diabetes^10,11^. The overexpression of *PPARD* was observed in various human cancers, such as colorectal, pancreatic and lung cancer^12–15^. Previous studies revealed that *PPARD* polymorphisms were associated with lipid levels, metabolic traits, obesity and risk of coronary heart diseases (CHD) and cancers^16–19^. *PPARD* rs2016520 is located in the 5′-untranslated region of exon, which has been widely studied in multiple physiological and pathological process^20–23^. However, little is known on the relationship of *PPARD* polymorphisms with glioma risk and prognosis.

*PPARG* is located in human chromosome 3p25 and encodes a nuclear receptor (PPARγ) activated by fatty acid metabolites or synthetic medicines^24–26^. *PPARG* is mainly expressed in suprabasal keratinocytes, adipocyte tissue, vascular endothelial cells, macrophage cells and smooth muscle cells^27,28^. *PPARG* regulates adipocyte differentiation and controls genes expression involved in lipid and glucose homeostasis^29^. And, *PPARG* has anti-inflammatory effect by restraining the production of inflammatory mediators^30^. It has been reported that *PPARG* is implicated in the pathology of obesity, diabetes, atherosclerosis and cancer. Wang *et al*. indicated that PPARG could arrest cell growth in human oral cancer^31^. Fan *et al*. pointed that anti- *PPARG* therapy is a potential strategy to improve endocrine-resistant breast cancer^32^. Nevertheless, the role of *PPARG* in glioma has not been elucidated.

Therefore, we conducted a case-control study to investigate the association of *PPARD* and *PPARG* polymorphisms (rs2016520, rs67056409, rs1053049, rs2206030, rs2920503, rs4073770 and rs1151988) with glioma risk and prognosis in the Chinese Han population.

## Methods

### Study population

This study consisted of 568 glioma patients and 509 healthy controls, recruited from the Second Affiliated Hospital of Xi’an Jiaotong University, Shaanxi Province, China. All glioma patients were newly diagnosed and histologically confirmed according to the World Health Organization (WHO) classification^33^. The exclusion criteria of glioma patients are as follows: (1) patients have history of cancer or CNS diseases; (2) patients are under 18 years old. The controls were healthy individuals without history of cancer or serious diseases who randomly enrolled from the same hospital. We obtained demographic and clinical information of study population from medical records and follow-up. This study was performed in accordance with the Declaration of Helsinki, and it was approved by the ethics committee of the Second Affiliated Hospital of Xi’an Jiaotong University. Informed consents were required form all participants before this study.

### SNP selection and genotyping

Combined previously studies, we selected four SNPs of *PPARD* (rs2016520, rs67056409, rs1053049 and rs2206030) and three SNPs of *PPARG* (rs2920503, rs4073770 and rs1151988), with minor allele frequencies (MAF) greater than 5% in the HapMap Chinese Han Beijing population. We extracted DNA from peripheral blood samples using the blood DNA kit (GoldMag Co. Ltd., Xiʹan, China). SNP genotyping was performed in the Agena MassARRAY system (Agena, San Diego, CA, USA). Primers for polymerase chain reaction (PCR) amplification and extension were designed by the Agena MassARRAY Assay Design 3.0 Software (San Diego, CA USA). PCR primers of selected SNPs were listed in Supplemental Table 1. In addition, we used Agena Typer 4.0 Software (San Diego, CA, USA) to manage and analyze data.

### Statistical analysis

We conducted all statistical analysis using Microsoft Excel and SPSS version 21.0 software (SPSS, Chicago, IL, USA). Student’s *t*-test and chi-square test were used to compare the differences in age and sex between glioma patients and healthy controls. The Hardy-Weinberg equilibrium (HWE) was checked for controls with Fisher’s exact test. We assessed the association of *PPARD* and *PPARG* polymorphisms with glioma risk by calculating odd ratios (OR) and 95% confidence intervals (CI) using logistic regression. Multifactor dimensionality reduction (MDR, version 3.0.2) was used to analyze SNP-SNP interactions on glioma risk. Then, we plotted patient survival curves by the Kaplan-Meier method and log-rank test. The association of *PPARD* and *PPARG* polymorphisms with OS and progression free survival (PFS) of glioma patients was evaluated by calculating hazard ratios (HR) and 95%CI using univariate and multivariate analysis. In multivariable survival analysis, we assessed the associations of *PPARD* and *PPARG* polymorphisms with glioma prognosis adjusted by age, sex, WHO grade, surgery, radiotherapy and chemotherapy. All tests were two-sided, and *P* < 0.05 was regarded as statistical significance. Additionally, our results were adjusted for multiple comparison using false discovery rate (FDR) correction.

## Results

### Characteristics of study population

The characteristics of 568 glioma patients and 509 healthy controls were presented in Table 1. The mean ages of the cases and controls were 39.68 ± 16.96 and 41.32 ± 15.69 years old, respectively. No significant variation in age or sex was found between the two groups (age: *P* = 0.102, sex: *P* = 1.000). Among glioma patients, 438 (77%) people were astrocytoma. According to WHO grading standards, 35 (6%) patients were grade I, 320 (56%) patients were grade II, and others are in high-grade glioma (III + IV). In addition, surgery method, radiotherapy and chemotherapy of patients were shown in Table 1.

**Table 1:** Comparison of glioma patients and controls by characteristics.

Characteristics	Glioma patients (N = 568)	Healthy controls (N = 509)	*P*
Age	39.68 ± 16.96	41.32 ± 15.69	0.102
>40	296(52%)	241(47%)	
≤40	272(48%)	268(53%)	
Sex			1.000
Male	313(55%)	280(55%)	
Female	255(45%)	229(45%)	
Astrocytoma			
Yes	438(77%)		
No	130 (23%)		
**WHO grade**			
I	35(6%)		
II	320(56%)		
III	149(26%)		
IV	64(12%)		
**Surgery**			
STR & NTR	181 (32%)		
GTR	387 (68%)		
**Radiotherapy**			
No	59 (10%)		
Conformable radiotherapy	154 (27%)		
Gamma knife	355 (63%)		
**Chemotherapy**			
No	337 (59%)		
Yes-temodar	49 (9%)		
Yes-not temodar	182 (32%)		

WHO, World Health Organization; STR, sub-total resection; NTR, near-total resection; GTR, gross-total resection.

### Association of *PPARD* and *PPARG* polymorphisms with glioma risk

In Table 2, *PPARD* and *PPARG* polymorphisms were accord with HWE in controls (*P* > 0.05).

**Table 2:** Association of *PPARD* and *PPARG* polymorphisms with glioma risk.

Gene	SNP	Chr: position	HaploReg v4.1	Group	Genotype			Allele frequency		Model	OR(95%CI)	*P*	FDR*-P*
					AA	AB	BB	MAF (A)	HWE- *P*				
PPARD	rs2016520	6: 35378778	SiPhy cons, Promoter histone marks, Enhancer histone marks, DNAse, Motifs changed, GRASP QTL hits, Selected eQTL	case	31	217	317	0.247		Allele	0.82(0.68–0.99)	**0.041**	0.214
				control	37	217	255	0.286	0.385	Co-dominant	0.67(0.40–1.11)	0.123	0.323
											0.80(0.62–1.03)	0.085	0.275
										Dominant	0.78(0.62–1.00)	**0.047**	0.214
										Recessive	0.74(0.45–1.21)	0.231	0.404
										Additive	0.81(0.67–0.99)	**0.037**	0.214
PPARD	rs67056409	6: 35383699	Promoter histone marks, Enhancer histone marks, DNAse, Motifs changed, Selected eQTL	case	32	226	310	0.255		Allele	0.82(0.68–1.00)	**0.046**	0.214
				control	40	219	250	0.294	0.455	Co-dominant	0.64(0.39–1.06)	0.081	0.275
											0.83(0.65–1.07)	0.147	0.323
										Dominant	0.80(0.63–1.02)	0.072	0.275
										Recessive	0.70(0.43–1.13)	0.146	0.323
										Additive	0.82(0.67–0.99)	**0.041**	0.214
PPARD	rs1053049	6: 35395618	DNAse, Motifs changed, GRASP QTL hits, Selected eQTL	case	30	203	334	0.232		Allele	0.78(0.64–0.95)	**0.012**	0.214
				control	39	206	264	0.279	1.000	Co-dominant	0.60(0.37–1.00)	0.051	0.214
											0.78(0.60–1.00)	0.051	0.214
										Dominant	0.75(0.59–0.96)	**0.020**	0.214
										Recessive	0.67(0.41–1.10)	0.114	0.323
										Additive	0.78(0.64–0.95)	**0.012**	0.214
PPARD	rs2206030	6: 35404354	Enhancer histone marks, Motifs changed, NHGRI/EBI GWAS hits, Selected eQTL	case	126	291	151	0.478		Allele	1.08(0.91–1.28)	0.371	0.546
				control	106	255	148	0.459	0.929	Co-dominant	1.17(0.83–1.65)	0.382	0.546
											1.12(0.84–1.48)	0.436	0.573
										Dominant	1.13(0.87–1.48)	0.361	0.546
										Recessive	1.08(0.81–1.45)	0.587	0.685
										Additive	1.08(0.91–1.29)	0.365	0.546
PPARG	rs2920503	3: 12324230	Motifs changed	case	55	233	280	0.302		Allele	1.00(0.83–1.20)	0.985	0.986
				control	43	221	245	0.302	0.529	Co-dominant	1.12(0.72–1.73)	0.612	0.695
											0.92(0.72–1.19)	0.529	0.635
										Dominant	0.95(0.75–1.21)	0.702	0.776
										Recessive	1.16(0.76–1.77)	0.482	0.595
										Additive	1.00(0.83–1.20)	0.986	0.986
PPARG	rs4073770	3: 12368233	Enhancer histone marks, Motifs changed, Selected eQTL	case	60	265	243	0.339		Allele	0.99(0.83–1.19)	0.924	0.972
				control	69	209	231	0.341	0.061	Co-dominant	0.83(0.56–1.22)	0.339	0.546
											1.21(0.93–1.56)	0.152	0.323
										Dominant	1.11(0.87–1.41)	0.390	0.546
										Recessive	0.75(0.52–1.09)	0.132	0.323
										Additive	0.99(0.83–1.18)	0.925	0.971
PPARG	rs1151988	3: 12511512	Enhancer histone marks, Motifs changed, GRASP QTL hits, Selected eQTL	case	7	133	428	0.129		Allele	0.84(0.66–1.07)	0.162	0.324
				control	9	135	365	0.150	0.488	Co-dominant	0.66(0.24–1.80)	0.418	0.566
											0.84(0.64–1.11)	0.217	0.396
										Dominant	0.83(0.63–1.09)	0.175	0.334
										Recessive	0.69(0.26–1.87)	0.470	0.595
										Additive	0.83(0.65–1.07)	0.154	0.323

SNP, single nucleotide polymorphism; MAF, minor allele frequency; HWE, Hardy-Weinberg equilibrium; OR, odds ratio; CI, confidence interval; FDR, false discovery rate.

Bold values indicate statistical significance (*P* < 0.05).

HaploReg (https://pubs.broadinstitute.org/mammals/haploreg/haploreg.php) predicted that PPARD and PPARG polymorphisms were related to the regulation of SiPhy cons, Promoter histone marks, Enhancer histone marks, DNAse, Motifs changed, GRASP QTL hits, NHGRI/EBI GWAS hits, Selected eQTL. After adjustment for age and sex, *PPARD* polymorphisms (rs2016520, rs67056409 and rs1053049) were significantly associated with glioma risk (*P* < 0.05). Rs2016520 and rs1053049 of *PPARD* had a decreased glioma risk in allele (rs2016520: OR = 0.82, 95%CI = 0.68–0.99, *P* = 0.041; rs1053049: OR = 0.78, 95%CI = 0.64–0.95, *P* = 0.012), dominant (rs2016520: OR = 0.78, 95%CI = 0.62–1.00, *P* = 0.047; rs1053049: OR = 0.75, 95%CI = 0.59–0.96, *P* = 0.020) and additive (rs2016520: OR = 0.81, 95%CI = 0.67–0.99, *P* = 0.037; rs1053049: OR = 0.78, 95%CI = 0.64–0.95, *P* = 0.012) models. We found that the allele distribution of rs67056409 were significantly different between cases and controls (*P* = 0.046), and subjects had lower risk of glioma in additive model (OR = 0.82, 95%CI = 0.67–0.99, *P* = 0.041). There were no significant association between glioma risk and other genetic polymorphisms (rs2206030, rs2920503, rs4073770 and rs1151988). However, FDR analysis revealed that the significant associations between genetic polymorphisms and glioma risk were not reliable.

We further did stratification analysis of *PPARD* and *PPARG* polymorphisms with glioma risk (Tables 3 and 4). For the subjects older than 40 years old, rs2016520, rs67056409 and rs1053049 of *PPARD* significantly decreased risk of glioma in multiple models (*P* < 0.05). Rs1053049 had a strong relationship with decreased risk of glioma in the subgroup of male (allele: OR = 0.76, 95%CI = 0.59–0.99, *P* = 0.041; dominant: OR = 0.71, 95%CI = 0.51–0.98, *P* = 0.040; additive: OR = 0.76, 95%CI = 0.59–0.99, *P* = 0.040). Then, we divided glioma patients to astrocytoma and others, we found that rs2016520, rs67056409 and rs1053049 of *PPARD* were significantly associated astrocytoma risk compared with other glioma (*P* < 0.05). We also explored the effect of WHO grade on the relationship of genetic polymorphisms with glioma risk. The results showed *PPARG* rs2920503 was strongly related to higher risk of high-grade glioma (III + IV) in co-dominant (OR = 2.04, 95%CI = 1.13–3.68, *P* = 0.018) and recessive (OR = 2.03, 95%CI = 1.15–3.57, *P* = 0.014) models. After FDR correction, the protective effects of rs2016520, rs67056409 and rs1053049 on glioma risk were still significant among the individuals older than 40 years old (FDR- *P* < 0.05).

**Table 3:** Association of *PPARD* and *PPARG* polymorphisms with glioma risk stratified by age and sex.

Gene	SNP	Model	Age						Sex					
			>40			≤40			Male			Female		
			OR(95%CI)	*P*	FDR*-P*	OR(95%CI)	*P*	FDR*-P*	OR(95%CI)	*P*	FDR*-P*	OR(95%CI)	*P*	FDR*-P*
PPARD	rs2016520	Allele	0.66(0.50–0.88)	**0.004**	**0.034**	1.01(0.77–1.31)	0.964	0.970	0.82(0.64–1.06)	0.130	0.455	0.82(0.61–1.09)	0.170	0.476
		Co-dominant	0.48(0.22–1.06)	0.070	0.173	0.90(0.45–1.79)	0.765	0.970	0.78(0.4–1.51)	0.460	0.855	0.54(0.24–1.19)	0.124	0.476
			0.61(0.43–0.88)	**0.008**	**0.047**	1.08(0.75–1.55)	0.675	0.970	0.74(0.53–1.04)	0.084	0.370	0.89(0.61–1.29)	0.526	0.757
		Dominant	0.60(0.42–0.84)	**0.004**	**0.034**	1.05(0.74–1.49)	0.774	0.970	0.75(0.54–1.03)	0.079	0.370	0.83(0.58–1.19)	0.312	0.624
		Recessive	0.59(0.27–1.28)	0.179	0.376	0.87(0.45–1.69)	0.678	0.970	0.89(0.47–1.70)	0.730	0.902	0.56(0.26–1.23)	0.149	0.476
		Additive	0.65(0.48–0.87)	**0.004**	**0.034**	1.01(0.76–1.33)	0.955	0.970	0.81(0.62–1.06)	0.119	0.454	0.81(0.60–1.09)	0.164	0.476
PPARD	rs67056409	Allele	0.65(0.49–0.86)	**0.003**	**0.034**	1.03(0.80–1.34)	0.811	0.970	0.80(0.62–1.03)	0.088	0.370	0.85(0.64–1.14)	0.276	0.580
		Co-dominant	0.40(0.19–0.85)	**0.017**	0.071	0.99(0.5–1.96)	0.969	0.970	0.73(0.38–1.41)	0.347	0.855	0.54(0.25–1.16)	0.112	0.476
			0.67(0.46–0.96)	**0.027**	0.095	1.12(0.78–1.6)	0.544	0.970	0.73(0.52–1.02)	0.069	0.370	0.97(0.67–1.41)	0.883	0.883
		Dominant	0.62(0.44–0.88)	**0.007**	**0.047**	1.10(0.78–1.55)	0.596	0.970	0.73(0.53–1.01)	0.058	0.370	0.90(0.63–1.29)	0.562	0.757
		Recessive	0.47(0.22–0.98)	**0.045**	0.135	0.93(0.48–1.82)	0.842	0.970	0.84(0.45–1.59)	0.595	0.855	0.54(0.26–1.15)	0.111	0.476
		Additive	0.65(0.49–0.86)	**0.003**	**0.034**	1.05(0.79–1.39)	0.732	0.970	0.79(0.61–1.03)	0.082	0.370	0.85(0.63–1.13)	0.264	0.580
PPARD	rs1053049	Allele	0.68(0.51–0.91)	**0.009**	**0.047**	0.89(0.68–1.17)	0.407	0.970	0.76(0.59–0.99)	**0.041**	0.370	0.80(0.60–1.08)	0.143	0.476
		Co-dominant	0.41(0.18–0.93)	**0.033**	0.107	0.80(0.41–1.57)	0.518	0.970	0.63(0.33–1.21)	0.163	0.527	0.56(0.25–1.25)	0.159	0.476
			0.71(0.50–1.03)	0.069	0.173	0.88(0.61–1.26)	0.473	0.970	0.73(0.52–1.02)	0.065	0.370	0.85(0.58–1.23)	0.385	0.703
		Dominant	0.67(0.47–0.95)	**0.024**	0.092	0.86(0.61–1.22)	0.406	0.970	0.71(0.51–0.98)	**0.040**	0.370	0.80(0.56–1.15)	0.232	0.573
		Recessive	0.47(0.21–1.05)	0.065	0.173	0.85(0.44–1.63)	0.625	0.970	0.72(0.39–1.36)	0.315	0.855	0.60(0.27–1.32)	0.204	0.536
		Additive	0.68(0.51–0.91)	**0.010**	**0.047**	0.89(0.67–1.17)	0.389	0.970	0.76(0.59–0.99)	**0.040**	0.370	0.80(0.59–1.08)	0.142	0.476
PPARD	rs2206030	Allele	1.04(0.82–1.33)	0.726	0.828	1.10(0.86–1.40)	0.446	0.970	1.09(0.87–1.37)	0.460	0.855	1.07(0.83–1.38)	0.605	0.757
		Co-dominant	1.11(0.68–1.81)	0.683	0.820	1.25(0.75–2.07)	0.391	0.970	1.19(0.74–1.91)	0.471	0.855	1.14(0.69–1.89)	0.613	0.757
			1.29(0.85–1.96)	0.232	0.424	0.97(0.65–1.44)	0.866	0.970	1.13(0.78–1.65)	0.516	0.855	1.10(0.72–1.69)	0.659	0.762
		Dominant	1.23(0.83–1.83)	0.304	0.532	1.04(0.71–1.51)	0.848	0.970	1.15(0.80–1.64)	0.448	0.855	1.11(0.74–1.67)	0.602	0.757
		Recessive	0.93(0.62–1.40)	0.735	0.828	1.27(0.82–1.98)	0.280	0.970	1.10(0.73–1.64)	0.653	0.885	1.07(0.70–1.63)	0.753	0.791
		Additive	1.06(0.83–1.35)	0.665	0.820	1.10(0.86–1.41)	0.459	0.970	1.09(0.87–1.38)	0.450	0.855	1.07(0.83–1.38)	0.606	0.757
PPARG	rs2920503	Allele	1.00(0.77–1.30)	0.996	0.996	1.01(0.78–1.30)	0.970	0.970	1.05(0.82–1.34)	0.717	0.902	0.95(0.72–1.25)	0.705	0.762
		Co-dominant	1.15(0.61–2.17)	0.671	0.820	1.07(0.57–1.98)	0.840	0.970	1.18(0.65–2.14)	0.583	0.855	1.05(0.55–2.00)	0.878	0.883
			0.91(0.64–1.30)	0.604	0.794	0.91(0.63–1.31)	0.612	0.970	0.99(0.71–1.39)	0.971	0.971	0.84(0.58–1.23)	0.368	0.703
		Dominant	0.95(0.67–1.33)	0.749	0.828	0.94(0.66–1.33)	0.710	0.970	1.02(0.74–1.41)	0.891	0.959	0.88(0.61–1.25)	0.469	0.757
		Recessive	1.20(0.65–2.22)	0.561	0.794	1.11(0.61–2.02)	0.722	0.970	1.18(0.67–2.09)	0.561	0.855	1.14(0.61–2.11)	0.685	0.762
		Additive	1.00(0.77–1.31)	0.996	0.996	0.98(0.75–1.28)	0.900	0.970	1.05(0.82–1.35)	0.715	0.902	0.95(0.72–1.25)	0.708	0.762
PPARG	rs4073770	Allele	0.94(0.73–1.21)	0.605	0.794	1.05(0.82–1.35)	0.689	0.970	1.07(0.84–1.37)	0.573	0.855	0.90(0.69–1.18)	0.449	0.757
		Co-dominant	0.79(0.45–1.40)	0.414	0.669	0.86(0.50–1.50)	0.599	0.970	1.05(0.62–1.80)	0.848	0.960	0.63(0.35–1.12)	0.118	0.476
			1.02(0.71–1.46)	0.930	0.976	1.40(0.97–2.04)	0.076	0.970	1.17(0.83–1.65)	0.371	0.855	1.25(0.85–1.83)	0.256	0.580
		Dominant	0.97(0.68–1.36)	0.843	0.908	1.25(0.88–1.78)	0.206	0.970	1.14(0.83–1.58)	0.415	0.855	1.07(0.75–1.54)	0.704	0.762
		Recessive	0.78(0.46–1.34)	0.372	0.625	0.73(0.43–1.23)	0.237	0.970	0.98(0.59–1.62)	0.925	0.971	0.56(0.33–0.97)	0.038	0.476
		Additive	0.93(0.72–1.20)	0.569	0.794	1.04(0.81–1.34)	0.736	0.970	1.07(0.84–1.36)	0.575	0.855	0.91(0.70–1.18)	0.455	0.757
PPARG	rs1151988	Allele	0.78(0.54–1.12)	0.170	0.376	0.91(0.66–1.28)	0.599	0.970	0.96(0.69–1.33)	0.799	0.932	0.72(0.50–1.03)	0.074	0.476
		Co-dominant	0.52(0.09–3.20)	0.482	0.750	0.80(0.23–2.75)	0.723	0.970	0.71(0.19–2.68)	0.610	0.855	0.61(0.13–2.76)	0.520	0.757
			0.78(0.52–1.17)	0.224	0.424	0.91(0.61–1.35)	0.645	0.970	0.99(0.68–1.45)	0.969	0.971	0.69(0.46–1.04)	0.076	0.476
		Dominant	0.77(0.52–1.14)	0.191	0.382	0.9(0.62–1.33)	0.602	0.970	0.97(0.67–1.41)	0.885	0.960	0.68(0.46–1.02)	0.065	0.476
		Recessive	0.55(0.09–3.38)	0.523	0.785	0.82(0.24–2.81)	0.751	0.970	0.71(0.19–2.68)	0.611	0.855	0.67(0.15–3.03)	0.602	0.757
		Additive	0.77(0.53–1.12)	0.171	0.376	0.91(0.64–1.28)	0.577	0.970	0.96(0.68–1.34)	0.793	0.932	0.71(0.49–1.03)	0.068	0.476

SNP, single nucleotide polymorphism; MAF, minor allele frequency; HWE, Hardy-Weinberg equilibrium; OR, odds ratio; CI, confidence interval; FDR, false discovery rate.

Bold values indicate statistical significance (*P* < 0.05).

**Table 4:** Association of *PPARD* and *PPARG* polymorphisms with glioma risk stratified by pathological classification and WHO grade.

Gene	SNP	Model	Astrocytoma VS. Other glioma			WHO grade (III + IV VS. I + II)		
			OR(95%CI)	*P*	FDR- *P*	OR(95%CI)	*P*	FDR- *P*
PPARD	rs2016520	Allele	0.79(0.64–0.97)	**0.025**	0.224	0.97(0.73–1.28)	0.810	0.989
		Co-dominant	0.60(0.34–1.05)	0.072	0.236	1.08(0.50–2.33)	0.844	0.989
			0.80(0.61–1.05)	0.103	0.254	0.98(0.68–1.41)	0.923	0.992
		Dominant	0.77(0.60–1.00)	**0.048**	0.224	0.99(0.70–1.41)	0.973	0.992
		Recessive	0.66(0.38–1.14)	0.135	0.315	1.09(0.51–2.32)	0.826	0.989
		Additive	0.79(0.64–0.97)	**0.027**	0.224	1.01(0.76–1.35)	0.956	0.992
PPARD	rs67056409	Allele	0.80(0.65–0.98)	**0.033**	0.224	0.93(0.70–1.22)	0.598	0.989
		Co-dominant	0.58(0.34–1.01)	0.054	0.227	0.80(0.36–1.77)	0.584	0.989
			0.84(0.64–1.10)	0.198	0.362	1.03(0.72–1.48)	0.863	0.989
		Dominant	0.80(0.62–1.03)	0.089	0.239	1.00(0.71–1.42)	0.992	0.992
		Recessive	0.63(0.37–1.08)	0.091	0.239	0.79(0.36–1.72)	0.554	0.989
		Additive	0.80(0.65–0.99)	**0.039**	0.224	0.97(0.73–1.29)	0.828	0.989
PPARD	rs1053049	Allele	0.76(0.61–0.93)	**0.009**	0.224	0.92(0.69–1.23)	0.581	0.989
		Co-dominant	0.56(0.32–0.98)	**0.043**	0.224	0.87(0.39–1.95)	0.744	0.989
			0.77(0.59–1.02)	0.064	0.236	0.97(0.67–1.40)	0.871	0.989
		Dominant	0.74(0.57–0.96)	**0.024**	0.224	0.96(0.67–1.36)	0.810	0.989
		Recessive	0.62(0.36–1.08)	0.091	0.239	0.89(0.40–1.95)	0.762	0.989
		Additive	0.76(0.62–0.94)	**0.012**	0.224	0.95(0.71–1.28)	0.754	0.989
PPARD	rs2206030	Allele	1.09(0.91–1.31)	0.342	0.497	1.08(0.85–1.38)	0.510	0.989
		Co-dominant	1.18(0.82–1.70)	0.376	0.497	1.14(0.69–1.88)	0.615	0.989
			1.09(0.81–1.48)	0.572	0.632	1.38(0.91–2.10)	0.131	0.523
		Dominant	1.12(0.84–1.49)	0.447	0.539	1.30(0.88–1.94)	0.192	0.620
		Recessive	1.12(0.82–1.52)	0.489	0.555	0.92(0.61–1.39)	0.684	0.989
		Additive	1.09(0.91–1.30)	0.372	0.497	1.08(0.84–1.38)	0.560	0.989
PPARG	rs2920503	Allele	0.91(0.75–1.12)	0.379	0.497	1.27(0.98–1.64)	0.074	0.499
		Co-dominant	0.92(0.57–1.49)	0.744	0.801	2.04(1.13–3.68)	**0.018**	0.378
			0.85(0.65–1.11)	0.235	0.386	1.02(0.70–1.47)	0.933	0.992
		Dominant	0.86(0.67–1.11)	0.255	0.397	1.17(0.83–1.65)	0.377	0.989
		Recessive	0.99(0.63–1.58)	0.982	0.982	2.03(1.15–3.57)	**0.014**	0.378
		Additive	0.91(0.75–1.12)	0.370	0.497	1.27(0.98–1.64)	0.074	0.499
PPARG	rs4073770	Allele	1.02(0.85–1.24)	0.818	0.838	0.80(0.62–1.03)	0.083	0.499
		Co-dominant	0.86(0.56–1.31)	0.480	0.555	0.70(0.38–1.27)	0.240	0.672
			1.28(0.98–1.69)	0.073	0.236	0.73(0.51–1.06)	0.095	0.499
		Dominant	1.18(0.91–1.53)	0.211	0.369	0.73(0.51–1.03)	0.071	0.499
		Recessive	0.76(0.51–1.13)	0.170	0.340	0.82(0.46–1.45)	0.489	0.989
		Additive	1.03(0.85–1.24)	0.799	0.838	0.80(0.61–1.04)	0.092	0.499
PPARG	rs1151988	Allele	0.83(0.64–1.08)	0.160	0.340	1.25(0.88–1.78)	0.209	0.627
		Co-dominant	0.62(0.21–1.88)	0.403	0.513	1.54(0.34–7.05)	0.580	0.989
			0.84(0.62–1.13)	0.239	0.386	1.34(0.90–2.00)	0.154	0.539
		Dominant	0.82(0.61–1.10)	0.191	0.362	1.35(0.91–2.00)	0.137	0.523
		Recessive	0.65(0.22–1.97)	0.449	0.539	1.43(0.31–6.52)	0.646	0.989
		Additive	0.83(0.63–1.08)	0.164	0.340	1.32(0.92–1.90)	0.137	0.523

SNP, single nucleotide polymorphism; MAF, minor allele frequency; HWE, Hardy-Weinberg equilibrium; OR, odds ratio; CI, confidence interval; FDR, false discovery rate.

Bold values indicate statistical significance (*P* < 0.05).

### MDR analysis

We used MDR analysis to assess the impact of the interaction among seven SNPs. The results obtained from MDR analysis for one- to seven- locus modes were presented in Table 5. A seven-locus model including polymorphisms of *PPARD* (rs2016520, rs67056409, rs1053049 and rs2206030) and *PPARG* (rs2920503, rs4073770 and rs1151988) was the best model of SNP-SNP interaction for glioma risk (cross-validation consistency = 10/10, accuracy = 0.660, sensitivity = 0.751, specificity = 0.570, *P* < 0.001).

**Table 5:** MDR analysis of SNP-SNP interaction.

Model	Bal. Acc. CV Training	Bal. Acc. CV Testing	CV Consistency	Accuracy	Sensitivity	Specificity	OR(95%CI)	*P*
rs1053049	0.538	0.513	8/10	0.537	0.593	0.481	1.35(1.06–1.74)	**0.017**
rs1053049, rs2206030	0.554	0.512	5/10	0.550	0.646	0.454	1.52(1.18–1.95)	**0.001**
rs2920503, rs1053049, rs2206030	0.573	0.471	2/10	0.566	0.554	0.578	1.70(1.33–2.18)	<**0.001**
rs2920503, rs4073770, rs2016520, rs2206030	0.602	0.500	7/10	0.597	0.646	0.548	2.22(1.72–2.85)	<**0.001**
rs2920503, rs4073770, rs1151988, rs2016520, rs2206030	0.630	0.500	5/10	0.624	0.601	0.646	2.76(2.14–3.55)	<**0.001**
rs2920503, rs4073770, rs1151988, rs67056409, rs1053049, rs2206030	0.658	0.506	10/10	0.650	0.751	0.550	3.68(2.82–4.80)	<**0.001**
rs2920503, rs4073770, rs1151988, rs2016520, rs67056409, rs1053049, rs2206030	0.668	0.4951	10/10	0.660	0.751	0.570	3.98(3.05–5.20)	<**0.001**

MDR, multifactor dimensionality reduction; SNP, single nucleotide polymorphism; CV, cross-validation; OR, odds ratio; CI, confidence interval.

Bold values indicate statistical significance (*P* < 0.05).

### Clinical factors and glioma prognosis

After obtained follow-up data of glioma patients, we investigated the impact of clinical factors on glioma prognosis (OS and PFS). As shown in Table 6, surgery and chemotherapy had significant correlations with OS and PFS of glioma (*P* < 0.05, FDR- *P* < 0.05). The prognosis of patients had gross-total resection (GTR) was better than those had sub-total resection (STR) or near-total resection (NTR) (OS: Log-rank *P* = 1.54E-07, HR = 0.63, 95%CI = 0.53–0.77, *P* = 1.88E-06, FDR- *P* = 1.32E-05; PFS: Log-rank *P* = 1.91E-09, HR = 0.59, 95%CI = 0.49–0.71, *P* = 6.78E-08, FDR- *P* = 4.75E-07). Additionally, glioma patients who undergone chemotherapy lived longer than those not (OS: Log-rank *P* = 1.38E-05, HR = 0.69, 95%CI = 0.58–0.83, *P* = 7.47E-05, FDR- *P* = 0.000261; PFS: Log-rank *P* = 0.005, HR = 0.79, 95%CI = 0.66–0.95, *P* = 0.011, FDR- *P* = 0.039). There were no significantly associations between other clinical factors (sex, age, WHO grade and radiotherapy) and glioma prognosis (*P* > 0.05).

**Table 6:** The impact of clinical factors on glioma patient OS and PFS.

Variables		Total	Event	OS				PFS					
				Log-rank *P*	SR (1-/3-year)	HR (95%CI)	*P*	FDR- *P*	Log-rank *P*	SR (1-/3-year)	HR (95%CI)	*P*	FDR- *P*
Sex	Male	313	278	0.379	0.328/0.082	1.08 (0.92–1.28)	0.420	0.490	0.268	0.200/0.096	1.09 (0.92–1.30)	0.321	0.321
	Female	255	229		0.310/0.094					0.155/0.089			
Age	<40	250	215	0.074	0.355/0.117	1.16 (0.97–1.38)	0.101	0.235	0.064	0.206/0.119	1.16 (0.97–1.38)	0.097	0.136
	≥40	317	291		0.290/0.067					0.159/0.072			
WHO grade	I + II	355	311	0.121	0.328/0.108	1.14 (0.95–1.36)	0.155	0.271	0.096	0.192/0.108	1.15 (0.96–1.37)	0.136	0.159
	III + IV	213	196		0.305/0.064					0.158/0.067			
Surgery	NTR & STR	181	178	**1.54E-07**	0.204/−	**0.63 (0.53–0.77)**	**1.88E-06**	**1.32E-05**	**1.91E-09**	0.016/−	**0.59 (0.49–0.71)**	**6.78E-08**	**4.75E-07**
	GTR	387	329		0.374/0.123					0.254/0.126			
Radiotherapy	No	59	48	0.438	0.441/−				0.118	0.200/−			0.136
Conformal radiotherapy		154	126		0.250/0.152	1.08 (0.78–1.51)	0.636	0.636		0.217/0.154	1.37 (0.97–1.94)	0.070	
	Gamma knife	355	333		0.330/0.055	1.17 (0.87–1.59)	0.303	0.424		0.163/0.058	1.31 (0.96–1.80)	0.090	0.136
Chemotherapy	No	337	315	**1.38358E-05**	0.276/0.029	**0.69 (0.58–0.83)**	**7.46734E-05**	**0.000261**	**0.005**	0.164/0.057	**0.79 (0.66–0.95)**	**0.011**	**0.039**
	Yes	231	192		0.384/0.151					0.205/0.155			

OS, overall survival; PFS, progression free survival; SR, survival rate; HR, hazard ratio; CI, confidence interval; WHO, World Health Organization; STR, sub-total resection; NTR, near-total resection; GTR, gross-total resection; FDR, false discovery rate.

Bold values indicate statistical significance (*P* < 0.05).

### Association of *PPARD* and *PPARG* polymorphisms with glioma prognosis

Then, we assessed the association of *PPARD* (rs2016520, rs67056409, rs1053049 and rs2206030) and *PPARG* (rs2920503, rs4073770 and rs1151988) polymorphisms with glioma prognosis. In Supplemental Table 2, univariate analysis did not show a strong relationship of *PPARD* and *PPARG* polymorphisms with glioma prognosis (*P* > 0.05). Moreover, we did not observe significantly association of *PPARD* and *PPARG* polymorphisms with OS and PFS of glioma patients (*P* > 0.05, Supplemental Table 3).

## Discussion

In this case-control study, we examined the association of *PPARD* and *PPARG* polymorphisms with glioma risk and prognosis in the Chinese Han population. After FDR correction, we found that *PPARD* polymorphisms were significantly associated with glioma risk, and the effects were dependent on age (*P* < 0.05, FDR- *P* < 0.05). Moreover, surgery method and chemotherapy had strongly effects on glioma prognosis (Log-rank *P* < 0.05, *P* < 0.05, FDR- *P* < 0.05).

PPARs are involved in the regulation of metabolic homeostasis, whose activity are controlled by fatty acid ligands^34^. After activation, PPARs heterodimerize with retinoid X receptors (RXRs) to affect the expression of downstream genes. It is reported that PPARs might had a functional crosstalk concerning the control of their expression^35^. Previous studies on the role of PPARs signaling in cancer mainly based on the availability of PPARs agonists and antagonists^36^. In brain tumor stem cells, PPARγ agonists inhibit cell growth and induce cell cycle arrest^37^. In mice, expression of PPARδ is related to prognosis and metastatic ability of breast cancer cells^38^. Polymorphisms of *PPARD* and *PPAR*G are associated with risk and prognosis of many diseases, including cardiovascular disease, diabetes, brain diseases, medulloblastoma and other cancers^39–41^. In our study, we firstly observed that *PPARD* polymorphisms (rs2016520, rs67056409 and rs1053049) were significantly associated with glioma risk. Similar association has been reported in colorectal cancer^39^. It suggests that *PPARD* polymorphisms could be involved in the susceptibility of glioma development. And, stratified analysis showed the effects of *PPARD* polymorphisms on glioma risk were age-dependent. It provides a scientific basis on individualized treatment of glioma. The effects of *PPARD* polymorphisms on glioma risk might related to SiPhy cons, Promoter histone marks, Enhancer histone marks, DNAse, Motifs changed, GRASP QTL hits, NHGRI/EBI GWAS hits, Selected eQTL. However, our results should be confirmed in further studies, including next-generation technology, PCR, western-blot analysis, etc.

Glioma is likely to have unfavorable prognosis caused by rapid proliferation and diffuse brain invasion. Despite surgery, chemotherapy and radiotherapy treatments improve, the prognosis of glioma remains poor^42^. Recent studies reported that some lipophilic molecules have antiproliferation and/or differentiation effects on glioma cells, and PPARs mediated some activities of these processes^43^. PPARγ has been observed in transformed neural cells of human and PPARγ agonist interferes with glioma growth and malignancy^43–45^. In this study, we firstly confirmed the effects of surgery method and chemotherapy on prognosis of glioma patients. Then, we explored the association of *PPARD* and *PPAR*G polymorphisms with OS and PFS of glioma patients. No significant associations were observed by univariate and multivariate analysis. It demonstrated that *PPARD* and *PPAR*G polymorphisms might not contribute the prognosis of glioma.

There are some limitations in the present study. First, we selected and genotyped several polymorphisms of *PPARD* and *PPAR*G, more genetic polymorphisms should be studied in the future. Second, we could not evaluate more factors on the association of genetic polymorphisms and glioma risk due to the limited sample size and information. Third, the molecular mechanisms of *PPARD* and *PPAR*G on glioma risk and prognosis are not elucidated in our study.

## Conclusion

In conclusion, we found genetic polymorphisms of *PPARD* were associated with glioma risk in the Chinese Han population, which suggests the role of *PPARD* in the carcinogenesis of glioma.

It provided information on exploring the mechanism and targeted therapy of glioma, it also promotes the development of precision medicine on glioma. Further studies in larger samples with more ethnic groups are needed to validate our results and explore the mechanism of *PPARD* and *PPAR*G in glioma.

## Supplementary information

Supplementary information.

## References

[1] Ostrom QT (2019). CBTRUS statistical report: primary brain and other central nervous system tumors diagnosed in the United States in 2012–2016. Neuro-oncology.

[2] Ferlay, J. *et al*. GLOBOCAN 2012 v1. 0, cancer incidence and mortality worldwide: IARC CancerBase No. 11. *Lyon, France: International agency for research on cancer* **2016** (2013).

[3] Chen W (2016). Cancer statistics in China, 2015. CA: a cancer J. clinicians.

[4] Ostrom QT, Gittleman H, Stetson L, Virk SM, Barnholtzsloan JS (2015). Epidemiology of gliomas. Cancer Treat. Res..

[5] Stupp R (2005). Radiotherapy plus concomitant and adjuvant temozolomide for glioblastoma. N. Engl. J. Med..

[6] Tan NS (2007). The nuclear hormone receptor peroxisome proliferator-activated receptor β/δ potentiates cell chemotactism, polarization, and migration. Mol. Cell. Biol..

[7] Lee C-H (2006). PPARδ regulates glucose metabolism and insulin sensitivity. Proc. Natl Acad. Sci..

[8] Schmuth M (2004). Peroxisome proliferator-activated receptor (PPAR)-β/δ stimulates differentiation and lipid accumulation in keratinocytes. J. Investig. Dermatology.

[9] Berger, J. & Moller, D. The mechanisms of action of ppars. *Annu Rev Med*. (2002). 10.1146/annurev.med.53.082901.104018 11818483

[10] Luquet S (2003). Peroxisome proliferator-activated receptor δ controls muscle development and oxidative capability. FASEB J..

[11] Wang YX (2004). Regulation of Muscle Fiber Type and Running Endurance by PPARδ. PLoS Biol..

[12] He T-C, Chan TA, Vogelstein B, Kinzler KW (1999). PPARδ is an APC-regulated target of nonsteroidal anti-inflammatory drugs. Cell.

[13] Gupta RA (2000). Prostacyclin-mediated activation of peroxisome proliferator-activated receptor δ in colorectal cancer. Proc. Natl Acad. Sci..

[14] Abdollahi A (2007). Transcriptional network governing the angiogenic switch in human pancreatic cancer. Proc. Natl Acad. Sci..

[15] Pedchenko TV, Gonzalez AL, Wang D, DuBois RN, Massion PP (2008). Peroxisome proliferator–activated receptor β/δ expression and activation in lung cancer. Am. J. respiratory Cell Mol. Biol..

[16] Seedorf U, Aberle J (2007). Emerging roles of PPARδ in metabolism. Biochimica et. Biophysica Acta -Molecular Cell Biol. Lipids.

[17] Tang L, Lü Q, Cao H, Yang Q, Tong N (2016). PPARD rs2016520 polymorphism is associated with metabolic traits in a large population of Chinese adults. Gene.

[18] Luo W (2016). A population association study of PPAR δ gene rs2016520 and rs9794 polymorphisms and haplotypes with body mass index and waist circumference in a Chinese population. Ann. Hum. Biol..

[19] Panza A (2014). Peroxisome proliferator-activated receptor γ-mediated induction of microRNA-145 opposes tumor phenotype in colorectal cancer. Biochim. Biophys. Acta.

[20] Jin-Fang S (2015). PPARD rs2016520 polymorphism affects repaglinide response in Chinese Han patients with type?2 diabetes mellitus. Clin. Exp. Pharmacology Physiol..

[21] Hautala AJ (2007). Peroxisome proliferator-activated receptor-δ polymorphisms are associated with physical performance and plasma lipids: the HERITAGE Family Study. Am. J. Physiol. Heart Circ. Physiol.

[22] Yan Z (2005). PPARdelta+ 294T/C gene polymorphism related to plasma lipid, obesity and left ventricular hypertrophy in subjects with metabolic syndrome. Zhonghua Xin Xue Guan Bing. Za Zhi.

[23] Skogsberg J (2003). Peroxisome proliferator activated receptor delta genotype in relation to cardiovascular risk factors and risk of coronary heart disease in hypercholesterolaemic men. J. Intern. Med..

[24] Desvergne Ba, Michalik L, Wahli W (2004). Be fit or be sick: peroxisome proliferator-activated receptors are down the road. Mol. Endocrinol..

[25] Straus DS, Glass CK (2007). Anti-inflammatory actions of PPAR ligands: new insights on cellular and molecular mechanisms. Trends immunology.

[26] Varga T, Czimmerer Z, Nagy L (2011). PPARs are a unique set of fatty acid regulated transcription factors controlling both lipid metabolism and inflammation. Biochimica et. Biophysica Acta -Molecular Basis Dis..

[27] Neve BP, Fruchart J-C, Staels B (2000). Role of the peroxisome proliferator-activated receptors (PPAR) in atherosclerosis. Biochemical pharmacology.

[28] Icre, G., Wahli, W. & Michalik, L. In *Journal of Investigative Dermatology Symposium Proceedings*. 30–35 (Elsevier). 10.1038/sj.jidsymp.5650007 17069008

[29] Calle EE, Kaaks R (2004). Overweight, obesity and cancer: epidemiological evidence and proposed mechanisms. Nat. Rev. Cancer.

[30] Hutter, S., Knabl, J., Andergassen, U. & Jeschke, U. The role of PPARs in placental immunology: a systematic review of the literature. *PPAR research* **2013** (2013). 10.1155/2013/970276 PMC3608350 23554810

[31] Wang H-Y (2016). Rosiglitazone elevates sensitization of drug-resistant oral epidermoid carcinoma cells to vincristine by G2/M-phase arrest, independent of PPAR-γ pathway. Biomedicine Pharmacotherapy.

[32] Fan P, Abderrahman B, Chai TS, Yerrum S, Jordan VC (2018). Targeting Peroxisome Proliferator-Activated Receptor γ to Increase Estrogen-Induced Apoptosis in Estrogen-Deprived Breast Cancer Cells. Mol. cancer therapeutics.

[33] Guo X (2019). Impact of ANXA5 polymorphisms on glioma risk and patient prognosis. J. neuro-oncology.

[34] Chang, W. H. & Lai, A. G. Pan-cancer mutational landscape of the PPAR pathway reveals universal patterns of dysregulated metabolism and interactions with tumor immunity and hypoxia. *bioRxiv*, 563676 (2019). 10.1111/nyas.14170 31215667

[35] Ding Y (2012). Gene–Gene Interaction between PPARδ and PPARγ Is Associated with Abdominal Obesity in a Chinese Population. J. Genet. genomics.

[36] Laganà A (2016). Pleiotropic actions of peroxisome proliferator-activated receptors (PPARs) in dysregulated metabolic homeostasis, inflammation and cancer: Current evidence and future perspectives. Int. J. Mol. Sci..

[37] Chearwae W, Bright J (2008). PPARγ agonists inhibit growth and expansion of CD133+ brain tumour stem cells. Br. J. Cancer.

[38] Wang X (2016). PPAR-delta promotes survival of breast cancer cells in harsh metabolic conditions. Oncogenesis.

[39] Rosales-Reynoso, M. *et al*. Protective role of+ 294 T/C (rs2016520) polymorphism of PPARD in Mexican patients with colorectal cancer. *Genet. Mol. Res* **16** (2017). 10.4238/gmr16019324 28128413

[40] Huang Y (2015). PPARD rs2016520 polymorphism and circulating lipid levels connect with brain diseases in Han Chinese and suggest sex-dependent effects. Biomedicine Pharmacotherapy.

[41] Ye H (2015). Positive association between PPARD rs2016520 polymorphism and coronary heart disease in a Han Chinese population. Genet. Mol. Res..

[42] Coras R (2007). The peroxisome proliferator-activated receptor-γ agonist troglitazone inhibits transforming growth factor-β–mediated glioma cell migration and brain invasion. Mol. Cancer Therapeutics.

[43] Benedetti E (2008). Biomolecular characterization of human glioblastoma cells in primary cultures: differentiating and antiangiogenic effects of natural and synthetic PPARγ agonists. J. Cell. Physiol..

[44] Benedetti, E., Galzio, R., D’Angelo, B., Ceru, M. P. & Cimini, A. PPARs in human neuroepithelial tumors: PPAR ligands as anticancer therapies for the most common human neuroepithelial tumors. *PPAR research* **2010** (2010). 10.1155/2010/427401 PMC2841252 20339586

[45] Grommes C (2006). Inhibition of *in vivo* glioma growth and invasion by peroxisome proliferator-activated receptor γ agonist treatment. Mol. pharmacology.

