# Supplementary material for: The Impact of PPARD and PPARG Polymorphisms on Glioma Risk and Prognosis
Source: Sci Rep. 2020 Mar 20;10:5140. doi: 10.1038/s41598-020-60996-2 (PMC7083928; doi:10.1038/s41598-020-60996-2)
Supplement: Supplementary file 1 — Supplementary information. [file 41598_2020_60996_MOESM1_ESM.docx]

The Impact of *PPARD* and *PPARG* Polymorphisms on Glioma Risk and Prognosis

Xiaoying Ding^1^, Xinsheng Han^1^, Haozheng Yuan^1^, Yong Zhang^1^, Ya Gao^2,#^

^1^Department of Anesthesia, The Second Affiliated Hospital of Xi’an Jiaotong University, Xi’an, Shaanxi 710004, China

^2^Department of Pediatric Surgery, The Second Affiliated Hospital of Xi’an Jiaotong University, Xi’an, Shaanxi 710004, China

#Corresponding author

Ya Gao

Tel: +86 13991131816

E-mail: gaoya0929666@163.com

Address: No. 157, Xiwu Road, Xi'an, Shaanxi, China

**Supplemental Table 1** PCR primers of selected SNPs

| **Gene** | **SNP** | **1^st^-PCRP** | **2^nd^-PCRP** | **UEP_DIR** | **UEP_SEQ** |
| --- | --- | --- | --- | --- | --- |
| PPARD | rs2016520 | ACGTTGGATGCATAGCTCTGGCATCGTCTG | ACGTTGGATGATCCTCTTCCTTGTCACTGC | F | GGCTGATGGGAACCA |
| PPARD | rs67056409 | ACGTTGGATGGGAAGTTGATGTTGCAGCTC | ACGTTGGATGAAAAGACTGAGTGCCTACCG | R | CCTCCAGGCTGCCTT |
| PPARD | rs1053049 | ACGTTGGATGAATCCTGCCAGCAGAGAGTG | ACGTTGGATGTGCCTGGTCCTCCCTCCCAA | F | gggttCCCAGCCCCTGCCCCTA |
| PPARD | rs2206030 | ACGTTGGATGTGGATTGGACACCCTGAGC | ACGTTGGATGAAAGGGAGGAGGCGATTTGC | R | aatAGGCCTGCTGGTGGG |
| PPARG | rs2920503 | ACGTTGGATGTTCTGGGACATTTTAATGG | ACGTTGGATGGCCTCTCAGTGGGTTTTTCG | F | CAATACATAGCTATATTATATTCTTGA |
| PPARG | rs4073770 | ACGTTGGATGTTTCCTTTCCCTAGGTAGAG | ACGTTGGATGCTACCCAAGAATCAAACAGTC | R | CATTTGTTAAAATCTCCTTTTTTTTT |
| PPARG | rs1151988 | ACGTTGGATGGATGATGTGCGTAGTACCTG | ACGTTGGATGAAAAGAAGCTCACGTCACGC | F | TGGCAAGAGTAGCTGGC |

PCR, polymerase chain reaction; SNP, single nucleotide polymorphism.

**Supplemental Table 2** Univariate analysis of *PPARD* and *PPARG* polymorphisms with glioma prognosis

| **Gene** | **SNP** | **Genotype** | **OS** | | | | **PFS** | | | |
| --- | --- | --- | --- | --- | --- | --- | --- | --- | --- | --- |
|  |  |  | **Log-rank *p*** | **SR (1-/3-year)** | **HR(95%CI)** | ***p*** | **Log-rank *p*** | **SR (1-/3-year)** | **HR(95%CI)** | ***p*** |
| PPARD | rs2016520 | CC | 0.588 | 0.161/0.097 | 1.20(0.81-1.77) | 0.357 | 0.748 | 0.097/- | 1.13(0.77-1.67) | 0.538 |
|  |  | CT |  | 0.335/0.076 | 0.99(0.83-1.19) | 0.952 |  | 0.155/0.077 | 0.98(0.82-1.18) | 0.861 |
|  |  | TT |  | 0.322/0.096 | 1.00 |  |  | 0.198/0.093 | 1.00 |  |
| PPARD | rs67056409 | GG |  | 0.188/0.063 | 1.25(0.86-1.82) | 0.250 |  | 0.063/- | 1.30(0.89-1.90) | 0.171 |
|  |  | GA | 0.398 | 0.310/0.076 | 1.07(0.89-1.28) | 0.479 | 0.315 | 0.161/0.084 | 1.04(0.86-1.24) | 0.708 |
|  |  | AA |  | 0.341/0.101 | 1.00 |  |  | 0.201/0.095 | 1.00 |  |
| PPARD | rs1053049 | CC |  | 0.167/0.100 | 1.15(0.77-1.70) | 0.499 |  | 0.100/- | 1.11(0.75-1.65) | 0.594 |
|  |  | CT | 0.746 | 0.328/0.072 | 0.99(0.83-1.19) | 0.933 | 0.729 | 0.145/0.073 | 0.97(0.80-1.16) | 0.709 |
|  |  | TT |  | 0.326/0.097 | 1.00 |  |  | 0.201/0.093 | 1.00 |  |
| PPARD | rs2206030 | CC |  | 0.357/0.097 | 1.20(0.97-1.48) | 0.098 |  | 0.206/0.082 | 1.04(0.81-1.34) | 0.767 |
|  |  | TC | 0.169 | 0.309/0.053 | 1.07(0.83-1.38) | 0.603 | 0.105 | 0.154/0.061 | 1.21(0.97-1.49) | 0.085 |
|  |  | TT |  | 0.308/0.146 | 1.00 |  |  | 0.196/0.146 | 1.00 |  |
| PPARG | rs2920503 | TT |  | 0.309/0.055 | 1.12(0.83-1.52) | 0.445 |  | 0.145/0.055 | 1.12(0.83-1.51) | 0.452 |
|  |  | CT | 0.487 | 0.361/0.095 | 0.95(0.79-1.14) | 0.584 | 0.385 | 0.189/0.091 | 0.93(0.78-1.12) | 0.463 |
|  |  | CC |  | 0.288/0.090 | 1.00 |  |  | 0.173/0.096 | 1.00 |  |
| PPARG | rs4073770 | TT |  | 0.275/0.090 | 1.04(0.77-1.41) | 0.782 |  | 0.127/0.091 | 1.05(0.78-1.42) | 0.754 |
|  |  | TA | 0.954 | 0.306/0.088 | 1.01(0.84-1.22) | 0.894 | 0.941 | 0.168/0.097 | 1.01(0.84-1.21) | 0.932 |
|  |  | AA |  | 0.346/0.089 | 1.00 |  |  | 0.198/0.083 | 1.00 |  |
| PPARG | rs1151988 | GG |  | 0.286/0.143 | 0.98(0.44-2.19) | 0.655 |  | 0.143/- | 1.09(0.49-2.45) | 0.830 |
|  |  | AG | 0.884 | 0.308/0.070 | 1.05(0.85-1.29) | 0.956 | 0.757 | 0.169/0.037 | 1.07(0.87-1.31) | 0.518 |
|  |  | AA |  | 0.324/0.093 | 1.00 |  |  | 0.180/0.100 | 1.00 |  |

SNP, single nucleotide polymorphism; OS, overall survival; PFS, progression free survival; SR, survival rate; HR, hazard ratio; CI, confidence interval.

**Supplemental Table 3** Multivariate analysis of *PPARD* and *PPARG* polymorphisms with glioma prognosis

| **Gene** | **SNP** | **Genotype** | **OS** | | **PFS** | |
| --- | --- | --- | --- | --- | --- | --- |
|  |  |  | **HR(95%CI)** | ***p*** | **HR(95%CI)** | ***p*** |
| PPARD | rs2016520 | CC | 1.16(0.79-1.72) | 0.452 | 1.07(0.73-1.59) | 0.725 |
|  |  | CT | 1.06(0.88-1.28) | 0.546 | 1.03(0.86-1.24) | 0.750 |
|  |  | TT | 1.00 |  | 1.00 |  |
| PPARD | rs67056409 | GG | 1.13(0.77-1.66) | 0.536 | 1.20(0.82-1.76) | 0.353 |
|  |  | GA | 1.11(0.92-1.34) | 0.263 | 1.07(0.89-1.29) | 0.466 |
|  |  | AA | 1.00 |  | 1.00 |  |
| PPARD | rs1053049 | CC | 1.15(0.77-1.71) | 0.505 | 1.13(0.76-1.68) | 0.557 |
|  |  | CT | 1.08(0.89-1.30) | 0.442 | 1.04(0.86-1.25) | 0.707 |
|  |  | TT | 1.00 |  | 1.00 |  |
| PPARD | rs2206030 | CC | 0.98(0.79-1.21) | 0.328 | 0.85(0.66-1.11) | 0.230 |
|  |  | TC | 0.88(0.68-1.14) | 0.838 | 0.99(0.80-1.23) | 0.936 |
|  |  | TT | 1.00 |  | 1.00 |  |
| PPARG | rs2920503 | TT | 1.17(0.87-1.59) | 0.306 | 1.19(0.88-1.61) | 0.259 |
|  |  | CT | 0.91(0.75-1.10) | 0.322 | 0.90 (0.75-1.09) | 0.270 |
|  |  | CC | 1.00 |  | 1.00 |  |
| PPARG | rs4073770 | TT | 1.03(0.76-1.40) | 0.840 | 1.02(0.75-1.39) | 0.899 |
|  |  | TA | 1.05(0.87-1.27) | 0.587 | 1.07(0.86-1.28) | 0.501 |
|  |  | AA | 1.00 |  | 1.00 |  |
| PPARG | rs1151988 | GG | 0.79(0.35-1.78) | 0.564 | 0.87(0.38-1.96) | 0.730 |
|  |  | AG | 0.92(0.74-1.13) | 0.400 | 0.95 (0.77-1.16) | 0.601 |
|  |  | AA | 1.00 |  | 1.00 |  |

SNP, single nucleotide polymorphism; OS, overall survival; PFS, progression free survival; SR, survival rate; HR, hazard ratio; CI, confidence interval.

*P* values were adjusted by age, sex, WHO grade, surgery, radiotherapy and chemotherapy.
